# Impact of concussion and severe musculoskeletal injuries on the onset of mental health symptoms in male professional rugby players: a 12-month study

**DOI:** 10.1136/bmjsem-2019-000693

**Published:** 2019-12-22

**Authors:** Özgür Kilic, Phil Hopley, Gino M M J Kerkhoffs, Mike Lambert, Evert Verhagen, Wayne Viljoen, Paul Wylleman, Vincent Gouttebarge

**Affiliations:** 1 Amsterdam UMC, Univ of Amsterdam, Department of Orthopaedic Surgery, Amsterdam Movement Sciences, Meibergdreef 9, Amsterdam, Noord-Holland, The Netherlands; 2 Amsterdam Collaboration on Health & Safety in Sports (ACHSS), Amsterdam UMC IOC Research Center of Excellence, Amsterdam, The Netherlands; 3 Cognacity, London, United Kingdom; 4 Institute of Sport and Exercise Health, London, United Kingdom; 5 Division of Surgery, UCL, London, United Kingdom; 6 Academic Center for Evidence based Sports medicine (ACES), Amsterdam Movement Sciences, Amsterdam, The Netherlands; 7 Division of Exercise Science and Sports Medicine, University of Cape Town, Cape Town, South Africa; 8 Amsterdam UMC, Vrije Universiteit Amsterdam, Department of Public and Occupational Health, Amsterdam Movement Sciences, de Boelelaan 1117, Amsterdam, The Netherlands; 9 South African Rugby Union (SARU), Cape Town, South Africa; 10 Vrije Universiteit Brussel, Brussel, Belgium

**Keywords:** mental, rugby, sports & exercise medicine, concussion, injuries

## Abstract

**Objective:**

This study explored the association between concussion or musculoskeletal injuries, and the onset of mental health symptoms (MHS) in male professional rugby players over a 12-month period.

**Methods:**

Observational prospective cohort study with three measurements over a follow-up period of 12 months. At baseline, 573 participants provided informed consent. A total of 327 male professional rugby players (62% forwards, 38% backs) completed all follow-up assessments at baseline, 6 months and 12 months. The mean (±SD) age, height and weight of the participants at baseline was 25.9 (±4.4) years, 184.9 (±8.7) cm and 101.5 (±14.6) kg, respectively. Number of musculoskeletal injuries and number of confirmed concussions were assessed through single questions. Symptoms of distress, anxiety/depression, sleep disturbance, adverse alcohol use and eating disorders were assessed using validated questionnaires.

**Results:**

Professional rugby players who sustained a concussion within 12 months of baseline were more likely to develop MHS with ORs ranging from 1.5 (95% CI 1.0 to 2.1) for distress to 2.0 (1.2 to 3.6) for adverse alcohol use. Players who sustained a severe injury within 12 months of baseline were more likely to develop symptoms anxiety/depression with an OR of 1.5 (1.1 to 2.0). There was no significant association in both groups for other MHS.

**Conclusions:**

Rugby players who sustained concussion or severe injuries are up to two times more likely to develop symptoms of distress, adverse alcohol use or anxiety/depression.

**What are the findings?**

- In male professional rugby, there is a significant association between the reporting of concussion at baseline and the occurrence of symptoms of distress and adverse alcohol use over a subsequent period of 12 months.
- The odds of developing symptoms of anxiety or depression is 1.5 (1.1 to 2.0) among players with severe musculoskeletal injuries at baseline compared with players without injury at baseline.

**How might it impact on clinical practice in the future?**

- These findings stress the need that rugby players and stakeholders (coaches, medical staff etc) be more vigilant for the adverse psychological impact of concussion.
- Proactive psychological support should be considered to rugby players who have been concussed as the effects of concussion on mental health are re-emphasised.

## Introduction

Rugby is a popular international sport with over 5 million players across more than 110 different countries.^1^ Being exposed to high physical and biomechanical loads during training and competition, rugby players are associated with a high risk of injury.^2–5^ Time-loss injuries resulting in a long lay-off period are considered as major adverse events for the career of a professional rugby player, sometimes even leading to early retirement.^1 2 6^ Known risk factors for these injuries are previous injury, excessive load, fatigue and unsafe behaviour.^1 2^ In professional football, psychological factors such as distress and anxiety have been identified as predictors for injuries.^2^ A logical assumption is that these psychological factors might also contribute to musculoskeletal injuries among professional rugby players.

Symptoms of distress, anxiety/depression or substance abuse/dependence—typically referred to as mental health symptoms (MHS)—have recently been shown to be prevalent among professional rugby players.^7 8^ Prevalence (4 weeks) of MHS ranged from 15% for adverse alcohol use to 30% for anxiety/depression.^7^ Studies among non-athletic populations show somewhat lower prevalence of anxiety/depression, from 17% to 21% in Denmark (occupational population), 25% in the Netherlands (general and occupational population, young male employees) and 13% to 19% in Australia (general population). Symptoms of distress in both young and older Australian working populations shows a prevalence range from 5% to 18%.^9–13^

Studies in professional football (one cross-sectional study and one prospective cohort study) have shown that MHS developed as a consequence of musculoskeletal injuries.^14 15^ Among rugby players, it has been shown that concussion was associated with the onset of depression.^16^ It therefore follows that musculoskeletal injuries as well as concussion might be related to the onset of MHS among professional rugby players.

Consequently, this study aimed to explore the association between musculoskeletal injuries or concussion, and the onset of MHS (distress, anxiety/depression, sleep disturbance, eating disorders, adverse alcohol use) in male professional rugby players over a 12-month period. The hypothesis is that professional rugby players who had sustained severe musculoskeletal injuries or concussion are more likely to develop MHS in the subsequent 12-month follow-up period.

## Methods

### Design and ethics

The present study was an observational prospective cohort study with three measurements (questionnaires) over a follow-up period of 12 months. This study was reported in accordance with the statement from STROBE (Strengthening the Reporting of Observational Studies in Epidemiology) and the Declaration of Helsinki.^17 18^

### Participants and setting

Participants fulfilled the following inclusion criteria: (1) being an active professional player in rugby (Union, League, Sevens); (2) being 18 years old or older; (3) being male; (4) being able to read and comprehend texts fluently in either English, French or Spanish. Professional rugby players were defined as players committing significant time to rugby training and competing at the highest and second highest professional rugby level. The International Rugby Players’ Association asked nine national players’ associations in Australia, England, France, Ireland, Italy, New Zealand, Pacific Islands (including Fiji, Samoa, Tonga), South Africa and Wales to assist in recruiting participants. In addition, the rugby unions from Argentina, Canada and USA were asked to assist in recruiting participants.

Sample size calculation used for descriptive purposes (prevalence and incidence) indicated that at least 196 participants were needed after follow-up (CI of 95%; precision of 5%) to reach an anticipated and assumed population prevalence of 15% of mental health symptoms.^19–21^ This sample size is sufficient for testing the relationship between the independent and dependent variables under study as sample size calculation indicated that at least 66 participants were needed (n>50+8m where m is the number of independent variables).^4^ Considering a response rate of 33% (analogous with the response rate achieved in other prospective studies among professional athletes) and hypothesising a loss to follow-up rate of no more than 20%, we needed to invite at least 765 potential participants.^20 21^

### Dependent variables: mental health symptoms

MHS were operationalised by assessing symptoms of distress, anxiety/depression, sleep disturbance, eating disorders and adverse alcohol use. Distress in the previous 4 weeks (baseline) and in the previous 6 months (follow-up) was measured using the Distress Screener (three items scored on a 3-point scale) which is based on the four-dimensional symptom questionnaire (4DSQ) (eg, “Did you recently suffer from worry?”).^22 23^ The 4DSQ, that is, Distress Screener in English, French and Spanish, has been validated for a recall period of up to several weeks (internal consistency: 0.6–0.7; test–retest coefficients: ≥0.9; criterion-related validity: area under ROC curve ≥0.79).^22 23^ A total score ranging from 0 to 6 was obtained by summing up the answers on the three items, a score of 4 or more indicating the presence of symptoms of distress.^22 23^

The 12-item General Health Questionnaire (GHQ-12) was used to assess psychological symptoms related to anxiety/depression in the previous 4 weeks (baseline) and in the previous 6 months (follow-up) (eg, “Have you recently felt under strain?”).^5^ The GHQ-12 in English, French and Spanish has been validated for a recall period of up to several weeks (internal consistency: 0.7–0.9; criterion-related validity: sensitivity ≥0.70, specificity ≥0.75, area under ROC curve ≥0.83).^24 25^ Based on the traditional scoring system, a total score ranging from 0 to 12 was calculated by summing the answers on the 12 items, with a score of 3 or more indicating the presence of symptoms of anxiety/depression (area under curve=0.88).^24 25^

Based on the (short form) Patient Reported Outcomes Measurement Information System (PROMIS), sleep disturbance in the previous 4 weeks (baseline) and in the previous 6 months (follow-up) was assessed through four single questions (eg, “Have you recently had problems sleeping?”) scored on a 5-point scale (from ‘not at all’ to ‘very much’).^26 27^ The PROMIS in English, French and Spanish has been validated for a recall period of up to several weeks (internal consistency: >0.9; construct validity: product–moment correlations ≥0.96) (for detailed information, see www.nihpromis.org). A total score ranging from 1 to 20 is obtained by summing the answers to the four questions, a score of 13 or more indicating the presence of symptoms of sleep disturbance.^26 27^

The Eating disorder Screen for Primary care (five items scored as ‘yes’ or ‘no’; ‘0’ for favourable answers, ‘1’ for unfavourable answers) was used as a screening instrument to detect eating disorders in the previous 4 weeks (baseline) and in the previous 6 months (follow-up) (eg, “In the past 4 weeks, were you satisfied with your eating patterns?”).^28^ The Eating disorder Screen for Primary care has been validated in several languages including English, French and Spanish (criterion-related validity: sensitivity 100%, specificity 0.71). A total score ranging from 0 to 5 is obtained by summing the answers on the five items, a score of 2 or more indicating the presence of eating disorders.^28^

Level of alcohol consumption at the present time (baseline) and in the previous 6 months (follow-up) was detected using the 3-item AUDIT-C (eg, “How many standard drinks containing alcohol do you have on a typical day?”).^29^ The AUDIT-C in English, French and Spanish has been validated for a recall period of up to several weeks (test–retest coefficients: 0.6–0.9; criterion-related validity: area under ROC curve 0.70–0.97).^30 31^ A total score ranging from 0 to 12 was obtained by summing the answers on the three items, a score of 5 or more indicating the presence of adverse alcohol use.^29^

### Independent variables: musculoskeletal injuries and concussion

Participants were asked to report through a single question whether they had sustained musculoskeletal injuries in the previous 12 months (baseline) and in the previous 6 months (follow-up; eg, “How many severe injuries (other than concussion) have you had in the past 6 months?”). In our study, severe injury (not a concussion) was defined as any physical complaint that is sustained by a player during a rugby match or rugby training, irrespective of the need for medical attention or time‐loss from rugby activities, and that leads to either training or competition absence for more than 28 days.^32^ It was explicitly explained to the rugby players that an injury (defined as stated above) was not a concussion (as explained below) and consequently concussion was not an injury.^7^ The number of confirmed concussions that occurred in the previous 12 months (baseline) and in the previous 6 months (follow-up) was explored through a single question (eg, for follow-up, “How many concussions (other than muscle or joint injuries) diagnosed by a medical professional did you have in the past 6 months (training and competition)?”).^33^ The definition of injury and concussion was included in the questionnaire. For these questions, participants were recommended to consult their team doctor.

### Procedures

A baseline and two follow-up electronic questionnaires were set up in English, French and Spanish (FluidSurveys), including all dependent and independent variables from the study. In addition, the following descriptive variables were added in the baseline questionnaire: age, height, body mass, duration of professional rugby career, field position (forward or back), level of play, level of education, other employment and family history of mental disorders. Each questionnaire took about 15 to 20 min to complete. Information about the study was sent via email to potential participants by the participating national associations. Players interested in participating in the study gave their informed consent and were given access to the baseline online questionnaire, which they were asked to complete within 2 weeks. At the end of the baseline questionnaire, participants could leave their email address and give their informed consent specifically for the follow-up online questionnaires. Follow-up questionnaires were sent per email 6 and 12 months later, being asked to complete them within 2 weeks. Reminders at baseline and follow-up were sent after 2 and 4 weeks. The responses to baseline and follow-up questionnaires were anonymised for reasons of privacy and confidentiality. The baseline and follow-up questionnaires were linked within participants through a unique code. Once completed, the electronic questionnaires were saved automatically on a secured electronic server that only the principal researcher could access. Players participated voluntarily in the study and did not receive any reward for their participation. Baseline questionnaires were distributed between March and August 2016.

### Statistical analyses

All data analyses were conducted with the statistical software IBM SPSS Statistics V.23.0 for Windows. Descriptive data analyses (mean, SD, frequency) were performed with all variables included in our study. To explore whether loss to follow-up was selective, we compared baseline characteristics (all descriptive variables) of non-responders and responders at follow-up by means of independent t-tests.^3^ Only data without missing values were used for analyses. Two statistical models were used: (1) univariate logistic regression analysis, (2) multivariate logistic analysis (adjusted separately and combined for respectively age and family history of mental disorder). In these models, new injuries/concussion or MHS reported over the course of the follow-up period were considered. OR and 95% CI were calculated. For this analysis, only participants without a given mental health symptom at baseline were included.

## Results

### Participants

**Figure 1:**
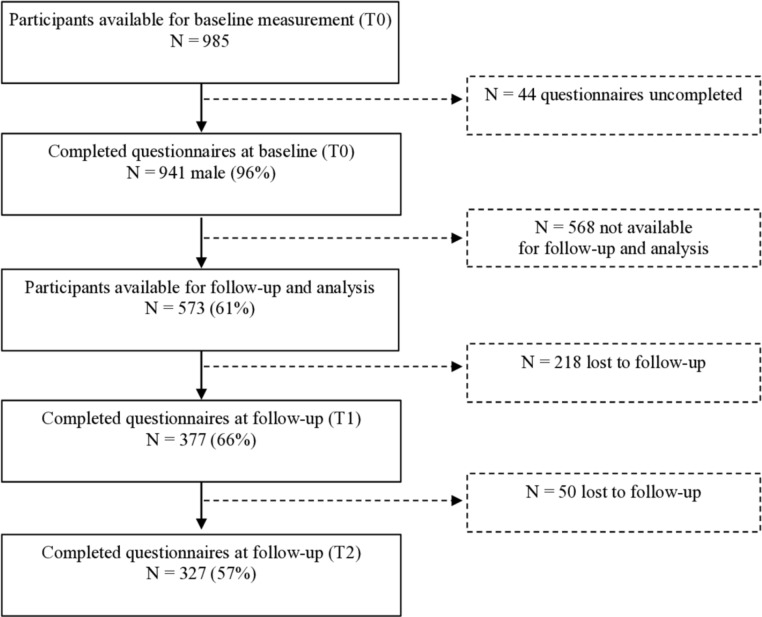
Flowchart of the recruitment and follow-up of male professional rugby players.

From the 941 participants that filled in the baseline questionnaire, 573 were included for analyses as they gave written informed consent to participate in the follow-up study. After the follow-up period of 1 year, a total of 327 players had completed both follow-up questionnaires. The flowchart of the recruitment of the participants is presented in figure 1. The mean±SD age, height and weight of the participants was 25.9±4.4 years, 184.9±8.7 cm and 101.5±14.6, respectively. Participants (62% forwards, 38% backs) had been playing professional rugby for an average of 6.0±4.0 years, with 74% playing at the highest club level in their country (mostly in Rugby Union). All characteristics of the participants are presented in table 1.

**Table 1:** Characteristics of the participants at baseline

	Total	No MHS	MHS
No of participants (%)	573	250 (44)	323 (56)
Age (years)	25.9±4.4	25.6±4.5	26.2±4.2
Height (cm)	184.9±8.7	184.9±8.7	185.0±8.8
Weight (kg)	101.5±14.6	100.5±14.0	102.1±15.1
Duration professional football career (years)	6.0±4.0	6.0±4.0	6.1±4.0
Level of play (highest level, %)	74	70	77
Field position %			
Forward	62	59	65
Back	38	41	35
International %	55	60	51
Educational level %			
No school completed	1	2	1
High school	39	37	41
Vocational/technical school	8	7	9
College, university or equivalent	51	54	48
Family history of mental health %	11	8	13
Baseline prevalence %			
Distress	20	–	35
Anxiety/depression	32	–	56
Sleep disturbance	12	–	22
Adverse alcohol use	15	–	27
Eating disorder	22	–	39
Injury	56	54	57
Concussion	35	28	40

Values are mean±SD unless otherwise stated.

Level of play (highest level, %) states only the percentage of players competing at the highest level (not second highest level and lower).

MHS, mental health symptoms.

### Associations between concussion and the onset of mental health symptoms

The odds of developing MHS in the 12-month follow-up period among concussed professional rugby players and were initially without MHS at baseline were significantly higher compared with players who had not sustained concussion at baseline (OR (95% CI) ranging from 1.5 (1.0 to 2.1) for distress to 2.0 (1.2 to 3.6) for adverse alcohol use). All analysed associations are shown in table 2.

**Table 2:** Association between concussion and the onset of MHS

	Univariate	Multivariate: adjusted for age	Multivariate: adjusted for family history	Multivariate: adjusted for age and family history
Association between a concussion reported on baseline and the onset of MHS during the subsequent 12 months				
Distress	1.7 (0.9 to 3.4)	1.5 (0.8 to 3.0)	1.7 (0.9 to 3.4)	1.5 (0.8 to 3.0)
Anxiety/depression	1.1 (0.6 to 2.0)	1.1 (0.6 to 2.1)	1.1 (0.6 to 2.1)	1.1 (0.6 to 2.1)
Sleeping disturbance	1.0 (0.5 to 1.9)	1.0 (0.5 to 1.9)	1.0 (0.5 to 1.9)	1.0 (0.5 to 1.9)
Adverse alcohol use	**2.0 (1.1 to 3.5)**	2.0 (1.1 to 3.5)	**2.0 (1.2 to 3.6)**	**2.0 (1.1 to 3.6)**
Eating disorder	1.1 (0.5 to 2.2)	1.1 (0.5 to 2.3)	1.1 (0.5 to 2.2)	1.1 (0.5 to 2.3)
Comorbidity	1.4 (0.8 to 2.6)	1.4 (0.8 to 2.5)	1.4 (0.8 to 2.6)	1.4 (0.8 to 2.5)
Association between the number of concussion recorded on baseline and the onset of MHS during the subsequent 12 months				
Distress	**1.5 (1.0 to 2.1)**	1.4 (0.9 to 2.0)	**1.5 (1.0 to 2.1)**	1.4 (0.9 to 2.0)
Anxiety/depression	1.1 (0.8 to 1.6)	1.1 (0.8 to 1.6)	1.1 (0.8 to 1.6)	1.1 (0.8 to 1.6)
Sleeping disturbance	1.0 (0.7 to 1.5)	1.0 (0.7 to 1.5)	1.0 (0.7 to 1.5)	1.0 (0.7 to 1.5)
Adverse alcohol use	**1.5 (1.1 to 2.0)**	**1.5 (1.1 to 2.0)**	**1.5 (1.1 to 2.1)**	**1.5 (1.1 to 2.1)**
Eating disorder	1.1 (0.6 to 1.7)	1.1 (0.7 to 1.7)	1.0 (0.6 to 1.7)	1.1 (0.7 to 1.7)
Comorbidity	1.3 (0.8 to 1.9)	1.2 (0.8 to 1.9)	1.3 (0.9 to 1.9)	1.2 (0.8 to 1.9)

In bold: significant results.

Values are OR (95% CI).

MHS, mental health symptoms.

### Association between musculoskeletal injuries and onset of mental health symptoms

Professional rugby players who had sustained severe musculoskeletal injuries and were without MHS at baseline, had higher odds (OR 1.5 (1.1 to 2.0)) of developing symptoms of anxiety or depression in the 12-month follow-up period, compared with players who had not sustained severe injuries. All other associations between injury at baseline and the onset of MHS in the subsequent 12 months were not significant. All analysed associations are shown in table 3.

**Table 3:** Association between musculoskeletal injury at baseline and the onset of MHS

	Univariate	Multivariate: adjusted for age	Multivariate: adjusted for family history	Multivariate: adjusted for age and family history
Association between a musculoskeletal injury at baseline and the onset of MHS during the subsequent 12 months				
Distress	1.2 (0.6 to 2.5)	1.2 (0.5 to 2.5)	1.2 (0.6 to 2.6)	1.2 (0.5 to 2.5)
Anxiety/depression	1.2 (0.6 to 2.5)	1.5 (0.7 to 2.8)	1.3 (0.7 to 2.5)	1.5 (0.8 to 2.9)
Sleeping disturbance	1.1 (0.5 to 2.2)	1.1 (0.5 to 2.3)	1.1 (0.5 to 2.2)	1.1 (0.5 to 2.3)
Adverse alcohol use	1.0 (0.5 to 1.9)	1.1 (0.6 to 2.1)	1.0 (0.6 to 2.0)	1.1 (0.6 to 2.1)
Eating disorder	0.6 (0.2 to 1.5)	0.6 (0.2 to 1.6)	0.6 (0.2 to 1.5)	0.6 (0.2 to 1.6)
Comorbidity	1.0 (0.5 to 1.9)	1.1 (0.5 to 2.1)	1.0 (0.5 to 2.0)	1.1 (0.5 to 2.1)
Association between the no of musculoskeletal injuries recorded at baseline and the onset of MHS during the subsequent 12 months				
Distress	1.0 (0.8 to 1.3)	1.0 (0.7 to 1.3)	1.0 (0.7 to 1.3)	1.0 (0.7 to 1.3)
Anxiety/depression	**1.5 (1.1 to 2.0)**	**1.5 (1.1 to 2.0)**	**1.5 (1.1 to 2.0)**	**1.5 (1.1 to 2.0)**
Sleeping disturbance	1.2 (1.0 to 1.5)*	1.2 (1.0 to 1.5)*	1.2 (1.0 to 1.5)*	1.2 (1.0 to 1.5)*
Adverse alcohol use	0.9 (0.7 to 1.2)	0.9 (0.7 to 1.2)	0.9 (0.7 to 1.2)	0.9 (0.7 to 1.2)
Eating disorder	0.9 (0.7 to 1.2)	0.9 (0.6 to 1.2)	0.9 (0.6 to 1.2)	0.9 (0.6 to 1.2)
Comorbidity	1.3 (1.0 to 1.6)*	1.3 (1.0 to 1.6)*	1.3 (1.0 to 1.6)*	1.3 (1.0 to 1.6)*

In bold: significant results.

Values are OR (95% CI).

*Suggest significance after rounded off to one decimal.

MHS, mental health symptoms.

## Discussion

Professional rugby players who sustained a concussion within 12 months of baseline were more likely to develop MHS compared with players without concussion, with OR ranging from 1.5 (95% CI 1.0 to 2.1) for distress to 2.0 (1.2 to 3.6) for adverse alcohol use. Players who sustained a severe injury within 12 months of baseline were more likely to develop symptoms anxiety/depression with an OR of 1.5 (1.1 to 2.0). There was no significant association in both groups for other symptoms of MHS.

### Comparison with other studies

Du Preez *et al* reported that rugby players with ≥3 concussions had greater odds for the onset of symptoms of depression compared with players with ≤2 concussions. They did not find a significant association between injuries and symptoms of depression.^16^ Within this study having had a concussion was associated with the onset of symptoms of alcohol abuse and symptoms of distress.

Earlier studies among athletes showed significant associations between severe musculoskeletal injuries and the onset of MHS in subsequent 12 months. Professional football players are almost 2 to 7 times more at risks for developing several MHS after severe injuries.^15^ Among professional Danish handball players, the odds for the onset of symptoms of alcohol abuse subsequent to severe injuries was up to 1.4.^34^ The odds for the onset of symptoms of distress subsequent to severe injuries was up to 1.3.^34^ These results are not totally in accordance with the results of this study as this study only shows an association of an OR of 1.5 between number of injuries and symptoms of anxiety or depression. All other associations with other MHS were not significant.

### Methodological considerations

MHS, injury and concussion were all measured through self-report, making it a limitation as one might logically assume that less subjective information could have been obtained if measurements were done through medical professionals. One potential source of bias might be that rugby players with a particular interest in MHS were more prone to participate. Another possible bias may arise from the ongoing taboo surrounding MHS among professional rugby players, just as is seen in other professional sports. This could have resulted in an underestimation in the extent of MHS.^7 35 36^ A limitation worth mentioning is that the follow-up rate in our study only reached 57%. We did not explore why participants withdrew from our study but potential reasons are that (1) the questionnaire might have been too time consuming for active professional rugby players during their ongoing season, and (2) the follow-up questionnaires were sent by the principal investigator and not by their players’ associations or unions (less commitment).

The validated questionnaires (only available in English, French and Spanish) were most likely not a limitation as players had to be able to read and comprehend fluently these three languages to be eligible to the study. The questionnaires concerning mental health symptoms in our study rely on a recall period up to 6 months. One might argue that monthly questionnaires would have generated more valid data than questionnaires with a 6-month recall period. Also worth mentioning is that there is no comparison group with the general population which would allow to make even more valid comparisons.

Strengths of this study are the acceptable response rate of 63% and an adequate follow-up rate of 55%, as it is suggested that follow-up rates from 50% are adequate.^37^ Another strength is the longitudinal design of the study among a large group of professional rugby players concerning a difficult topic.^8^

### Further implications for future studies

While this study demonstrates the association between concussion and MHS, the exact nature of the association is worthy of further exploration. Future studies could focus on the aspects of concussion and the number of concussions which may contribute to MHS (eg, physical symptoms such as pain; cognitive impairment; removal from training/playing; impact on self-esteem; level of peer and family support provided to injured players; mental health education for players/stakeholders; provision of preventative mental health interventions such as counselling/mindfulness practice etc).

In other athlete populations, besides severe musculoskeletal injuries, also adverse life events and lack of social support were identified as factors contributing to the onset of MHS.^38^ Furthermore, in an earlier study among rugby players, 95% of the participants stated that MHS could negatively influence the performance and 46% stated that specific support measures concerning this matter were not available in professional rugby.^39^ Consequently, identifying factors contributing to the onset of MHS following concussions or injury may inform the development of a multidisciplinary strategy involving both physical and psychological rehabilitation in order to prevent worsening MHS and guide decisions to ensure optimal but safe return to sports and performance.

### Clinical relevance of the findings and perspective

From a clinical perspective, the results emphasise the potential adverse psychological impact of concussion and severe musculoskeletal injuries among rugby players. The association between concussion or injuries and MHS re-emphasise the need to provide a pre-emptive multidisciplinary approach including psychological expertise to tackle symptoms of distress or anxiety and depression and reduce the likelihood of adverse alcohol use (and other maladaptive coping strategies) among concussed and injured rugby players.
